# From the Nathan Shock Centers Coordinating Center: Biology of Aging for Nonbiologists

**DOI:** 10.1093/geroni/igaa057.2979

**Published:** 2020-12-16

**Authors:** Steven Austad

## Abstract

Advances in the biology of aging that promise to dramatically extend the healthy length of human life are now appearing at a breathtaking rate. At the same time, baseless claims of research breakthroughs in maintaining youth have exploded on electronic media. It often difficult for people outside basic aging research to keep track of what is real, what is imaginary, progress. This symposium brings together four scientists working in some of the most exciting research areas to update attendees in terms that will be understandable to anyone. Our speakers were carefully selected both for their scientific achievements and for their outstanding skill as science communicators. Matt Kaeberlein (University of Washington) will discuss some of the most exciting emerging pharmacological interventions that delay or prevent multiple features of aging. Nathan LeBrasseur (Mayo Clinic) will update us on a specific type of pharmacological intervention – senolytics – which target aged, nonfunctioning cells distributed throughout the body to broadly improve and extend health. Morgan Levine (Yale) will discuss how newly discovered molecular clocks give us more accurate information on our real biological age than does our birth certificate and will explain the research significance of these clocks. Melissa Harris (UAB) will update us on progress in stem cell biology. The promise of stem cells to treat and possibly reverse multiple aspects of aging are real, but perhaps no area of research has been more abused by hyperbolic claims. Dr. Harris will separate the real hope from the false hype.

